# MAP3K8 (TPL2/COT) Affects Obesity-Induced Adipose Tissue Inflammation without Systemic Effects in Humans and in Mice

**DOI:** 10.1371/journal.pone.0089615

**Published:** 2014-02-24

**Authors:** Dov B. Ballak, Peter van Essen, Janna A. van Diepen, Henry Jansen, Anneke Hijmans, Tetsuya Matsuguchi, Helmut Sparrer, Cees J. Tack, Mihai G. Netea, Leo A. B. Joosten, Rinke Stienstra

**Affiliations:** 1 Department of Medicine, Radboud University Medical Centre, Nijmegen, The Netherlands; 2 Institute for Genomic and Metabolic Disease, Radboud University Medical Centre, Nijmegen, The Netherlands; 3 Nijmegen Institute for Infection Inflammation and Immunity, Radboud University Medical Centre, Nijmegen, The Netherlands; 4 Department of Oral Biochemistry, Field of Developmental Medicine, Kagoshima University, Graduate School of Medical and Dental Sciences, Sakuragaoka, Kagoshima, Japan; 5 Novartis Pharma AG, Basel, Switzerland; 6 Department of Human Nutrition, Wageningen University and Research Centre, Wageningen, The Netherlands; Institute for Nutritional Sciences, China

## Abstract

Chronic low-grade inflammation in adipose tissue often accompanies obesity, leading to insulin resistance and increasing the risk for metabolic diseases. MAP3K8 (TPL2/COT) is an important signal transductor and activator of pro-inflammatory pathways that has been linked to obesity-induced adipose tissue inflammation. We used human adipose tissue biopsies to study the relationship of MAP3K8 expression with markers of obesity and expression of pro-inflammatory cytokines (IL-1β, IL-6 and IL-8). Moreover, we evaluated obesity-induced adipose tissue inflammation and insulin resistance in mice lacking MAP3K8 and WT mice on a high-fat diet (HFD) for 16 weeks. Individuals with a BMI >30 displayed a higher mRNA expression of MAP3K8 in adipose tissue compared to individuals with a normal BMI. Additionally, high mRNA expression levels of IL-1β, IL-6 and IL-8, but not TNF -α, in human adipose tissue were associated with higher expression of MAP3K8. Moreover, high plasma SAA and CRP did not associate with increased MAP3K8 expression in adipose tissue. Similarly, no association was found for MAP3K8 expression with plasma insulin or glucose levels. Mice lacking MAP3K8 had similar bodyweight gain as WT mice, yet displayed lower mRNA expression levels of IL-1β, IL-6 and CXCL1 in adipose tissue in response to the HFD as compared to WT animals. However, MAP3K8 deficient mice were not protected against HFD-induced adipose tissue macrophage infiltration or the development of insulin resistance. Together, the data in both human and mouse show that MAP3K8 is involved in local adipose tissue inflammation, specifically for IL-1β and its responsive cytokines IL-6 and IL-8, but does not seem to have systemic effects on insulin resistance.

## Introduction

Obesity is characterized by chronic low-grade inflammation arising from the adipose tissue [1]. This inflammatory trait mainly results from resident or infiltrating immune cells into the adipose tissue and is associated with insulin resistance and metabolic diseases such as type 2 diabetes mellitus [2]. In response to pro-inflammatory stimuli, immune receptors activate signalling pathways, such as protein kinase like IκB kinase (IKK) and extra-cellular signal-regulated kinase (ERK). Stimulation of these pathways leads to activation of NF-κB and JNK transcription factors, resulting in transcription of pro-inflammatory genes including TNF-α, IL-6, IL-1β, and CCL2 [3]. These pathways have been recognized to play a pivotal role in instigating a local inflammatory reaction in the adipose tissue of obese patients, secondarily affecting the insulin signalling pathway [4]–[6].

Serine threonine mitogen activated protein kinase kinase kinase 8 (MAP3K8), in mice also called tumor progression locus 2 (TPL2) and in humans called Cancer Osaka Thyroid (COT), activates ERK-1/2 [7], [8]. In quiescent state, MAP3K8 forms a complex with A20-binding inhibitor of NF-κB (ABIN-2) and p105 NF-κB, precursor of the NF-κB transcription factor. It can be activated by pro-inflammatory stimuli, such as TNF-α, IL-1β and LPS. MAP3K8 knockout mice that are exposed to LPS/D-galactosamine-induced pathology are protected against endotoxin shock, showing that MAP3K8 is an essential protein in directing inflammatory responses [9]. The role of MAP3K8 in regulating the inflammatory trait of obesity is not fully clear. The function of MAP3K8 in obesity-induced inflammation has been studied previously.

One study reported that MAP3K8 is upregulated in adipose tissue in response to IL-1ß and TNF-α and mediates lipolysis induced by these cytokines [10]. Another study reported that MAP3K8 regulates obesity-associated inflammation and insulin resistance. MAP3K8 deficient mice showed a reduction of high fat diet (HFD)-induced adipose tissue inflammation and a reduced expression of inflammatory markers, as well as improved insulin sensitivity [11]. These results were not confirmed in a study that found contradictory results after conducting a similar high fat diet intervention study. The authors showed that MAP3K8 deficient mice were not protected against the detrimental effects of diet-induced obesity [12]. No differences in mRNA levels of several markers of adipose tissue inflammation or whole body glucose or insulin tolerance were observed in mice. Moreover, MAP3K8 was not up-regulated in adipose tissue due to HFD-feeding.

Considering these contradictory data in the literature, we aimed to illuminate the role of MAP3K8 using a complementary approach combining murine studies with assessment of the role of MAP3K8 in human adipose tissue. We found that human MAP3K8 expression in adipose tissue is indeed associated with obesity. However, using mice lacking MAP3K8, our data show a redundant role for MAP3K8 in obesity-associated metabolic dysfunction. Local adipose tissue inflammation was only mildly influenced. Moreover, human adipose tissue biopsies show that MAP3K8 expression in adipose tissue associates with mRNA levels of IL-1β, IL-6 and IL-8, but not with systemic metabolic parameters. Together these data suggest that MAP3K8 partially affects pro-inflammatory gene expression in adipose tissue, yet does not play an important role in the development of insulin resistance during obesity.

## Material and Methods

### Human subjects

Subcutaneous adipose tissues were obtained from 70 healthy donors subjects with a broad range of BMI. The measurements were carried out in the first and last quartile. The group was divided in low BMI and high BMI (BMI <25, n = 33, BMI >30, n = 18), low and high plasma insulin levels (concentration <5 mU/L, n = 22, >8 mU/L, n = 28); low and high plasma glucose levels (concentration <5 mM, n = 30, >5 mM, n = 40); low and high HOMA-IR levels (<2, n = 40, >2 n = 26); adipocyte cell size (diameter in μM, smallest and largest quartile n = 36). HOMA-IR was calculated by: (glucose * insulin plasma levels)/22.5. For association with mRNA levels of IL-1β, IL-6, IL-8 and TNFα to MAP3K8 lowest and highest quartile were compared (n = 36). Similarly, highest en lowest quartile of serum amyloid A (SAA) and C-reactive protein (CRP) levels were associated with MAP3K8 expression (n≥30) (SAA: Q1≤0.7 mg/L, Q4≥1.6 mg/L; CRP. Q1≤0.5 mg/L, Q4≥2.0 mg/L). All subjects gave written informed consent. The study was approved by the ethical committee of the Radboud University Medical Centre, Nijmegen.

### Animals

MAP3K8-ko mice [13] and WT mice on a C57Bl/6 background were housed in a pathogen-free environment in the animal facility from the Radboud University Nijmegen. All animal procedures were conducted under protocols approved by the animal experimentation committee of Radboud University Nijmegen Medical Centre. Bodyweight of the animals was recorded weekly. After a 2 weeks run-in period on low fat diet, mice were given low fat diet (LFD) or high fat diet (HFD) feeding for 16 weeks, containing 10% or 45% of energy derived from palm oil fat (D125450B or 12451; Research Diets, Inc).

### Oral glucose and insulin tolerance tests

Oral glucose tolerance (OGTT) and insulin tolerance tests (ITT) were performed. Prior to the OGTT, animals were fasted overnight (9 hours) and 2 g/kg glucose (D-glucose, Gibco, Invitrogen) was orally administered. Prior to the ITT, mice were fasted 6 hours and insulin (0,75 U/kg) was injected intraperitoneally. Blood glucose levels were determined with an Accu-chek glucosemeter (Roche Diagnostics, Almere, The Netherlands) at indicated time points after glucose administration.

### Histochemistry

For detection of macrophages/monocytes, an F4/80^+^ antibody (product code: MCA497G, AbD Serotec, Düsseldorf, Germany) was used for mice samples, a CD68-monoclonal antibody (Clone EBM11, Dako, Denmark) was used for human samples. Visualization of the complex was done using 3,3′-diaminobenzidene for 5 min. Negative controls were used by omitting the primary antibody. Morphometry of individual fat cells was assessed using digital image analysis. Microscopic images were digitized in 24-bit RGB (specimen pixel size 1.28×1.28 μm^2^). Recognition of fat cells was initially performed by applying a region-growing algorithm on manually indicated seed points, and minimum Feret diameter was calculated.

### qPCR

Total RNA was isolated from adipose tissue using TRIzol (Invitrogen, Carlsbad, CA), according to manufacturer's instructions. RNA was reverse-transcribed (iScript cDNA Synthesis Kit, Bio-Rad Laboratories). RT-PCR was performed using specific primers (see Table S1), power SYBR green master mix (Applied Biosystems, Foster City, CA) using the Step-one Real-Time PCR system (Applied Biosystems, Foster City, CA). For mice samples, we used both 36B4 and GAPDH as housekeeping genes. For human samples we used B2M as a housekeeping gene.

### Western blot analysis

Lysis buffer (50 mM Tris (pH 7.4), 150 mMNaCl, 2 mMEDTA, 1% Nonidet P-40, 50 mMNaF, and 0.25% sodium deoxycholate) with phosstop phosphatase-inhibitor cocktail tablet (Roche) and complete, EDTA-free protease-inhibitor cocktail tablet (Roche) was used to prepare adipose tissue lysates. The homogenized lysate was then centrifuged at 4°C for 10 min at 18.000 rcf. Subsequently, the supernatant was used for Western Blot. Equal amounts of protein as determined by a BCA protein assay (Thermo FisherScientific, Rockford, IL) were loaded, and separated using a polyacrylamide SDS page gel. After SDS-PAGE, proteins were transferred to a nitrocellulose membrane using Trans-Blot Turbo Transfer System (Biorad) following manufacturer's instructions. The membrane was blocked for 1 h at room temperature with 5% (wt/vol) milk powder in Tris-buffered saline (TBS)/Tween 20. Subsequently, the membrane was incubated overnight at 4°C with a phospho-p65 (Cell Signaling, 93H1) or pan-p65 antibody (Cell Signaling, D14E12), phospho-ERK (Promega, V8031) or pan-ERK antibody (Promega, V1141) and a tubulin antibody (Santa Cruz Biotechnology, 2–28–33) in 5% (wt/vol) milk powder/TBS/0.1% Tween 20. Hereafter, the blots were incubated with horseradish peroxidase-conjugated secondary antibodies (dilution of 1∶5000) in 5% (wt/vol) milk powder in TBS/0.1% Tween-20 for 1 h at room temperature and subsequently developed with Clarity reagent (Biorad) according to the manufacturer's instructions. Bands were visualized using a ChemiDoc System (Biorad) and quantified using Image lab software (Biorad).

### Plasma proteins

Plasma concentrations of insulin (ultra sensitive mouse insulin ELISA kit, Crystal Chem Inc., IL, USA; detection limit: 5 pg/ml) were measured by ELISA according to the manufacturer's instructions. Mice CXCL-1 concentrations were determined according to manufacturer's instructions (Duoset, R&D systems, MN, USA; detection limit 16 pg/ml). High sensitive C-reactive protein (hsCRP) was measured by enzyme-immunoassay according to the instructions from the manufacturer (Dako, Glastrup, Denmark; detection limit 3.1 ng/ml). SAA was measured using the N Latex SAA test (Siemens Healthcare Diagnostic, Germany, detection limit: 0.02 ng/ml) according to the instructions from the manufacturer.

### Plasma glucose

Glucose (Liquicolor, Human GmbH, Wiesbaden, Germany) was measured enzymatically following manufacturers' protocols.

### Statistical analysis

Data are shown as means ± SEM. Differences between groups were analyzed using Student's *t* test, differences among 4 groups were analyzed with ANOVA followed by *post-hoc* Bonferroni tests in Graphpad Prism 5.0. p-values <0.05 were considered significant.

## Results

### BMI, IL-6 and IL-8 expression are associated with higher MAP3K8 expression in human adipose tissue

**Figure 1:**
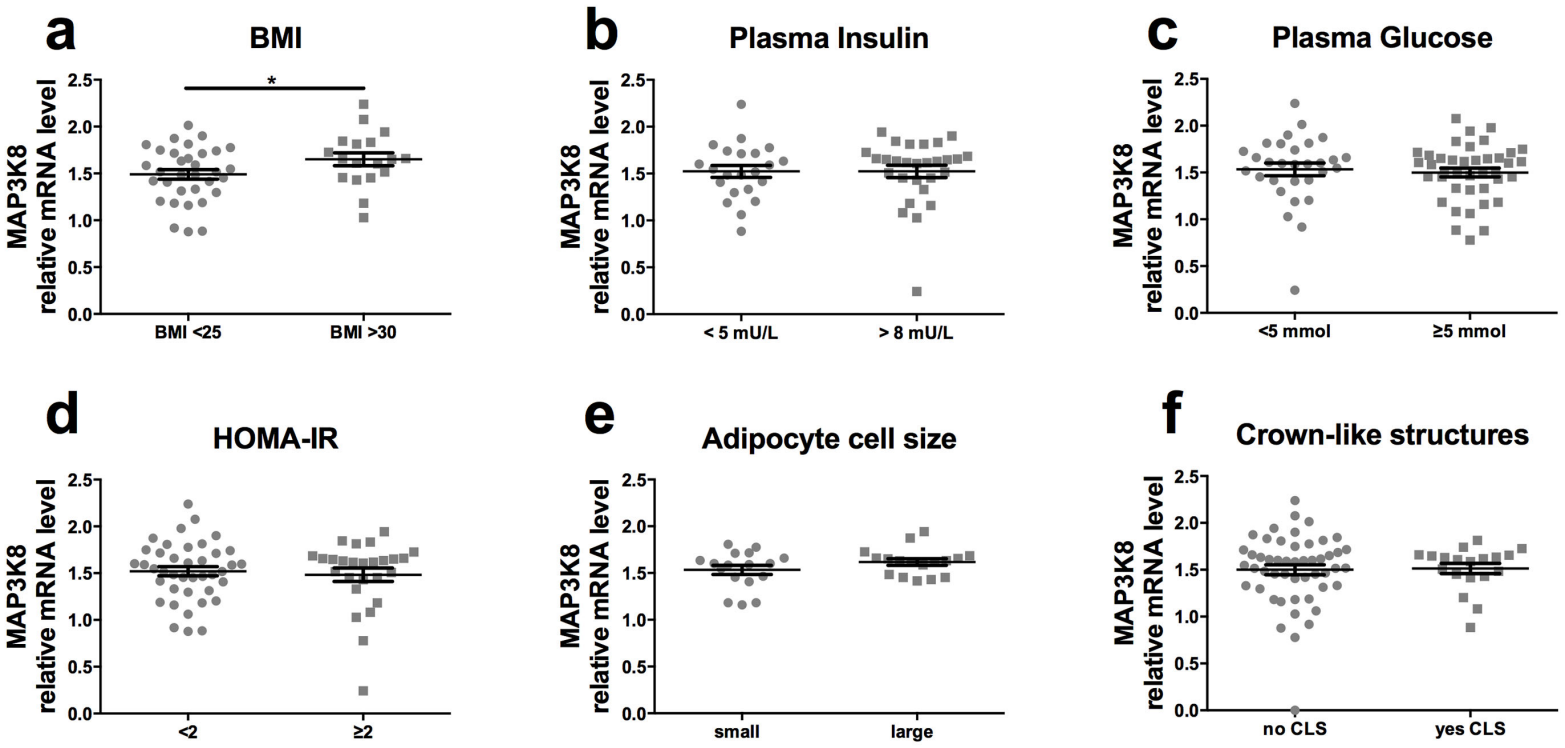
MAP3K8 in humans is associated with higher BMI and cytokine expression. MAP3K8 mRNA expression in human subcutaneous adipose tissue, associated with (a) BMI, (b) plasma insulin values, (c) plasma glucose levels, (d) HOMA-IR, (e) adipocyte sell size cell size and (f) crown-like structures. *p<0.05. n = 51, 50, 71, 70 respectively. HOMA-IR  =  Homeostatic Model Assessment for insulin resistance.

First, we determined the association of MAP3K8 (TPL2/COT) expression in human adipose tissue with measures of obesity (BMI), adipose tissue inflammation (cytokine expression) and insulin resistance (plasma insulin). Human subcutaneous tissue biopsies were acquired from healthy subjects with a wide range in BMI. As shown in **Figure 1a**, mRNA expression levels of MAP3K8 were significantly higher in individuals with a BMI higher than 30, compared to subjects with a normal BMI (between 20–25 kg/m^2^). However, no differences in MAP3K8 expression were observed between persons with low versus high plasma insulin and glucose levels (**Fig. 1b** **/c**). In line with this, no differences were observed for MAP3K8 expression in subjects with increased insulin resistance, as calculated by the homeostatic model for insulin resistance (**Fig. 1d**). Moreover, we did not see an increased expression of MAP3K8 in subjects with small versus large adipocyte size (**Fig. 1e**) or in subjects with crown-like structures (CLS) formed by infiltrating CD68 positive macrophages in adipose tissue compared to subjects with no CLS (**Fig. 1f**).

**Figure 2:**
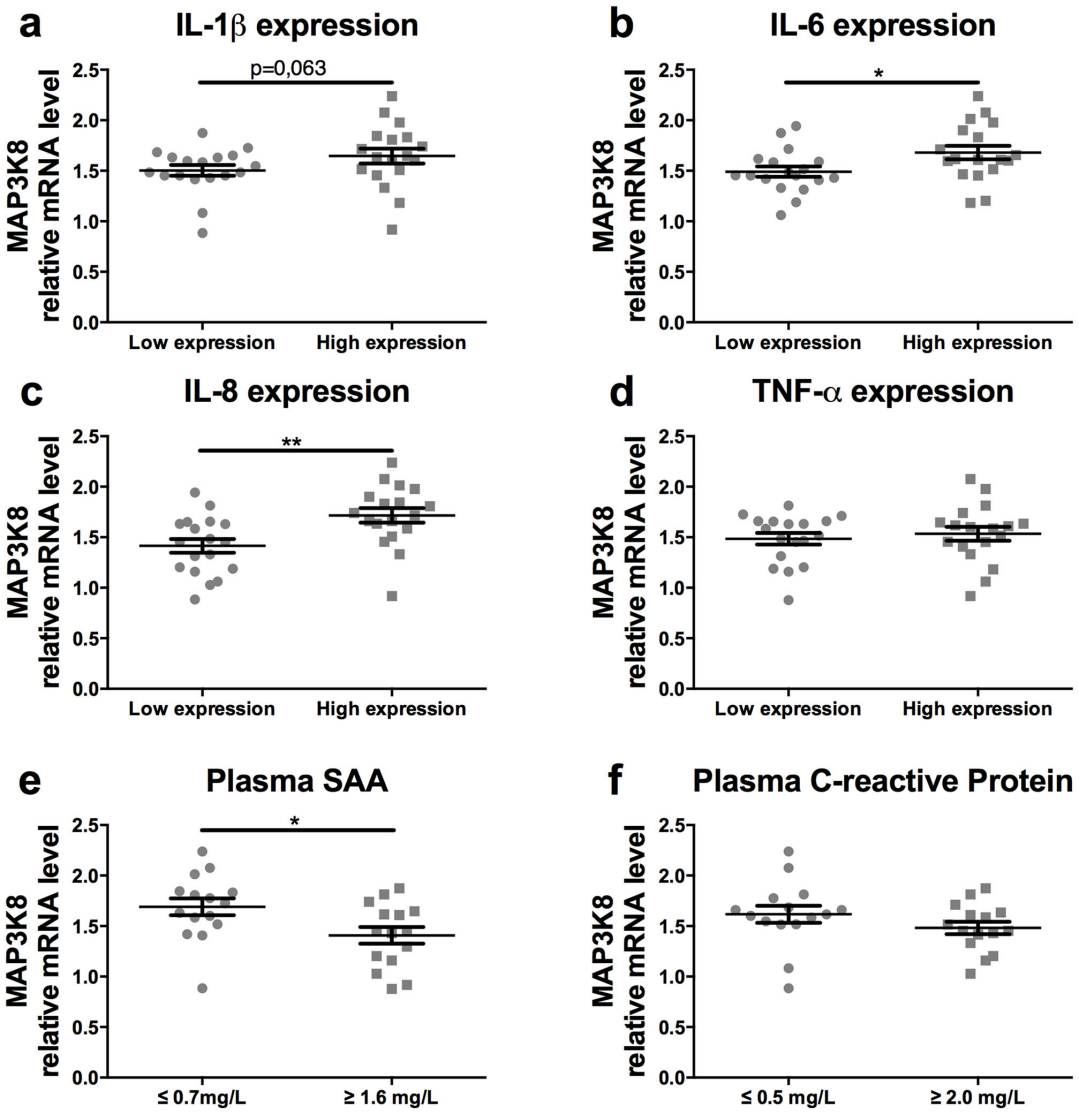
MAP3K8 in humans is associated with IL-1β, IL-6 and IL-8 cytokine expression. Biopsies from subcutaneous adipose tissue were obtained from healthy subjects with varying levels of obesity. Association of MAP3K8 mRNA expression in human subcutaneous adipose tissue with mRNA expression of (a) IL-1ß, (b) IL-6, (c) IL-8, (d) TNF-α, (e) serum amyloid A levels (SAA: Q1≤0.7 mg/L, Q4≥1.6 mg/L), (f) C-reactive protein (CRP: Q1≤0.5 mg/L, Q4≥2.0 mg/L). *p<0.05, **p<0.01.

Interestingly, we found that higher mRNA expression of IL-1ß in human adipose tissue was associated with higher mRNA levels of MAP3K8, although this difference did not reach statistical significance, p = 0.063 (**Fig. 2a**). Moreover, mRNA expression levels of IL-1ß responsive cytokines, IL-6 and IL-8, were significantly associated with higher MAP3K8 expression (**Fig. 2b** **/c**). In contrast, TNFα mRNA levels did not associate with MAP3K8 expression (**Fig. 2d**). Moreover, systemic inflammatory markers were measured in the plasma and related to MAP3K8 expression in adipose tissue. Higher levels of serum amyloid A (SAA) were negatively associated with MAP3K8 expression in adipose tissue (**Fig. 2e**). In contrast, no relation was measured for MAP3K8 expression and plasma C-reactive protein (CRP) levels (**Fig. 2f**).

### MAP3K8-ko mice show similar body weight compared to WT mice

**Figure 3:**
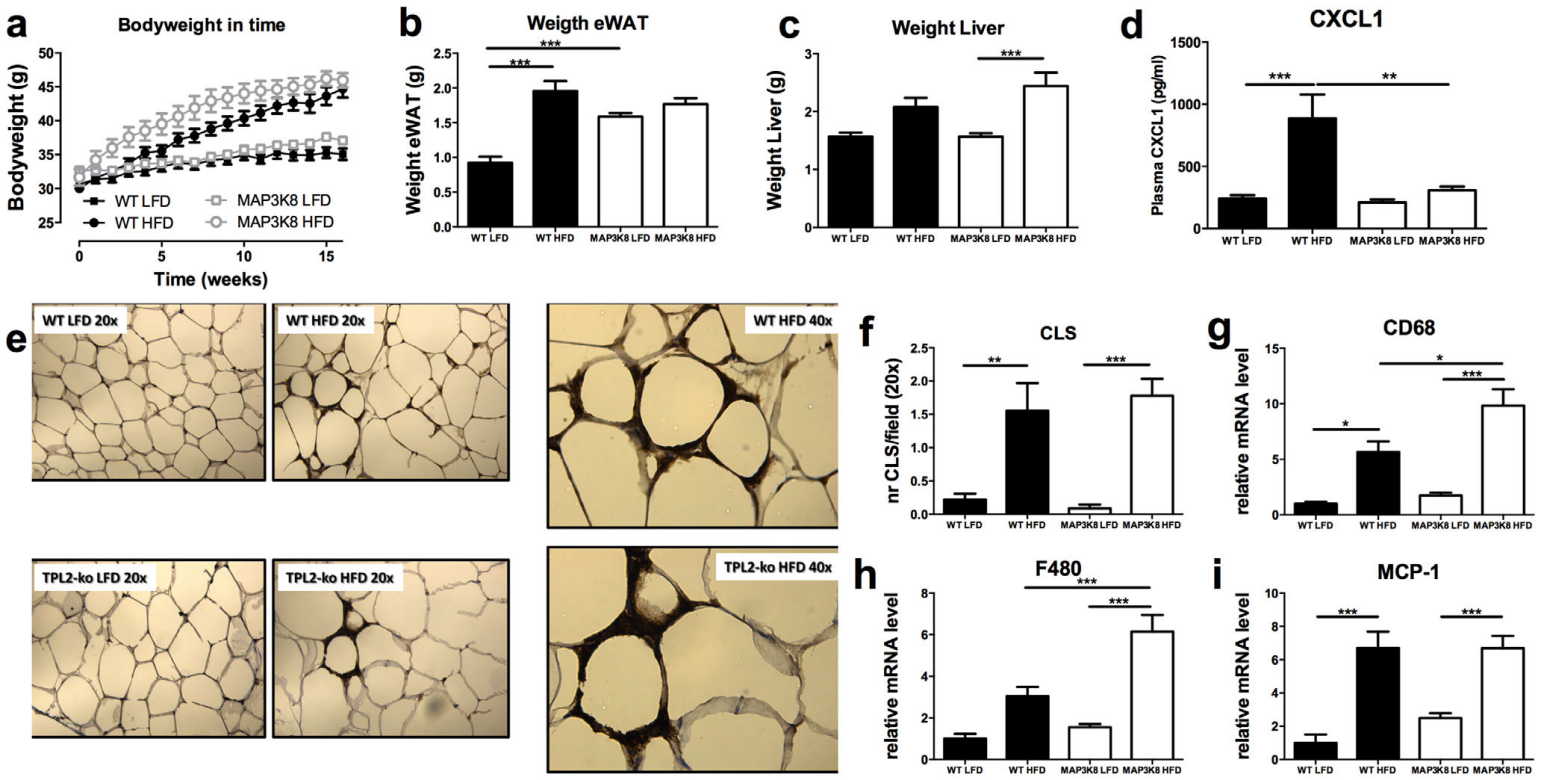
Obesity and macrophage influx in adipose tissue of HFD-fed WT and MAP3K8-ko animals. MAP3K8-ko and WT mice were fed a LFD or HFD during 16 weeks. (a) Bodyweight development upon LFD or HFD feeding. (b) Epididymal white adipose tissue (eWAT) weight after 16 weeks of LFD or HFD. (c) Liver weight after 16 weeks of LFD or HFD. (d) Plasma CXCL1 levels after 16 weeks of LFD or HFD (e) Macrophage influx into the adipose tissue as determined by immunohistochemistry, F4/80 (serotec) staining: 20× magnification or 40× as indicated: (f) Number of crown-like structures per field. (g–i) qPCR analysis for macrophage infiltration markers, (g) CD68, (h) F4/80, (i) MCP-1 in adipose tissue of MAP3K8-ko and WT animals. * p<0.05, ** p<0.01, *** p<0.001.

Based on the association of higher MAP3K8 expression with both BMI and enhanced levels of IL-1ß responsive genes in human adipose tissue, we set out to determine if MAP3K8 causally affects obesity and adipose tissue inflammation in vivo. Therefore, 12-week old MAP3K8 deficient or WT mice, were fed a high-fat diet (HFD, 45% calories by energy content derived from fat) or low-fat diet (LFD, 10%) for 16 weeks. Although MAP3K8-ko mice gained bodyweight faster during the initial phase of the diet intervention, at the end of the study there were no differences in bodyweight between MAP3K8-ko and WT mice (**Figure 3a**). At the end of the intervention period, both genotypes gained approximately 9–10 grams more bodyweight due to the HFD-feeding, compared to the LFD intervention. Moreover, MAP3K8-ko and WT mice had a similar epididymal white adipose tissue (eWAT) and liver weight after HFD feeding (**Fig. 3b** **/c**). Interestingly, MAP3K8-ko mice had a significant higher eWAT weight after LFD.

### Different mRNA expression profile of inflammatory in adipose tissue

Next, we investigated whether MAP3K8-deficiency affected inflammation in response to HFD feeding. Indeed, in WT mice systemic CXCL-1 levels were increased after HFD-feeding. However, this effect was blunted in MAP3K8ko mice (**Fig. 3d**). Adipose tissue sections were stained for F4/80 and the amount of crown-like structures (CLS) in the adipose tissue was counted (**Fig. 3e**). HFD feeding significantly increased the amount of CLS in adipose tissue similarly, in both MAP3K8-ko and WT mice (**Fig. 3f**). Interestingly, mRNA expression levels of the macrophage markers CD68 and F4/80 were significantly higher in MAP3K8-ko mice, while expression of MCP-1 was similar between both genotypes upon HFD feeding (**Fig. 3g–i**).

**Figure 4:**
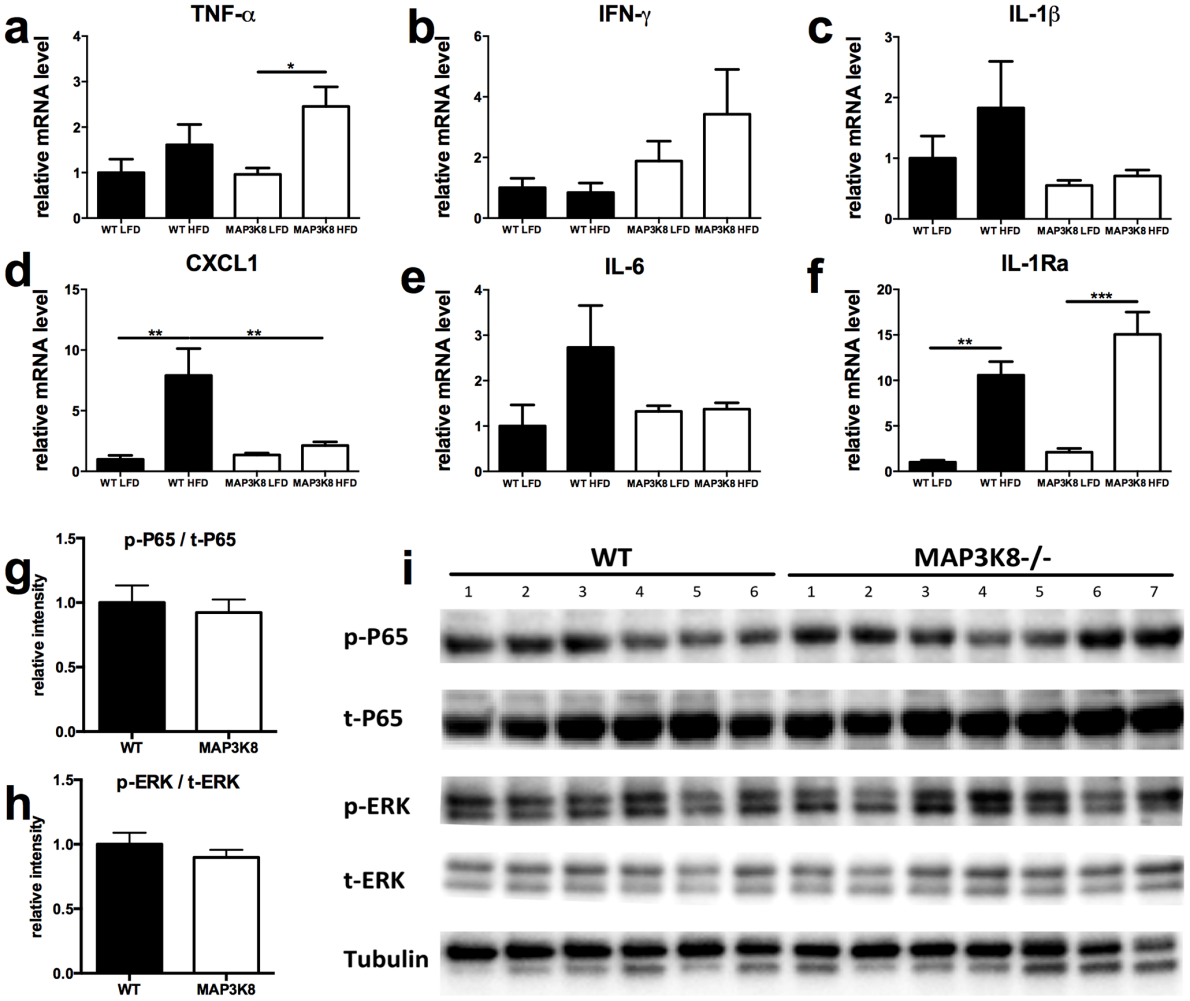
Inflammatory profile of the adipose tissue of HFD-fed WT and MAP3K8-ko animals. MAP3K8-ko and WT mice were fed a LFD or HFD during 16 weeks. (a–f) qPCR analysis for cytokines (a) TNF-α, (b) IFNγ, (c) IL-1β, (d) CXCL-1, (e) IL-6 and (f) IL-1Ra. n = 9 mice per group. Relative phosphorylation of NFκB p65 (g) and ERK 1/2 (h) in eWAT of MAP3K8-ko and WT animals after HFD-feeding (i). * p<0.05, ** p<0.01, *** p<0.001.

In line with an increased expression of macrophage markers, TNF-α and IFNγ mRNA levels tended to be increased in MAP3K8-ko mice fed a HFD, although this change did not reach statistical significance for IFNγ (**Fig. 4a** **/b**). In contrast, HFD-feeding did not upregulate expression of IL-1β and IL-1 effector cytokines IL-6 and CXCL-1 in MAP3K8-ko mice (**Fig. 4c–e**). Furthermore, mRNA IL-1Ra levels were not changed in MAP3K8-ko mice as compared to WT animals (**Fig. 4f**). To determine whether intracellular downstream targets of inflammatory pathways were affected in MAP3K8-ko versus WT mice, western blots were performed to measure the presence of phosphorylated ERK1/2 and NF-κB p65 in adipose tissue of both genotypes after HFD-feeding. No changes were observed in relative phosphorylation of both ERK1/2 and NF-κB p65 (**Fig. 4g** **/h**) as shown in **Figure 4i**.

### MAP3K8-ko and WT display similar glucose and insulin tolerance after HFD

**Figure 5:**
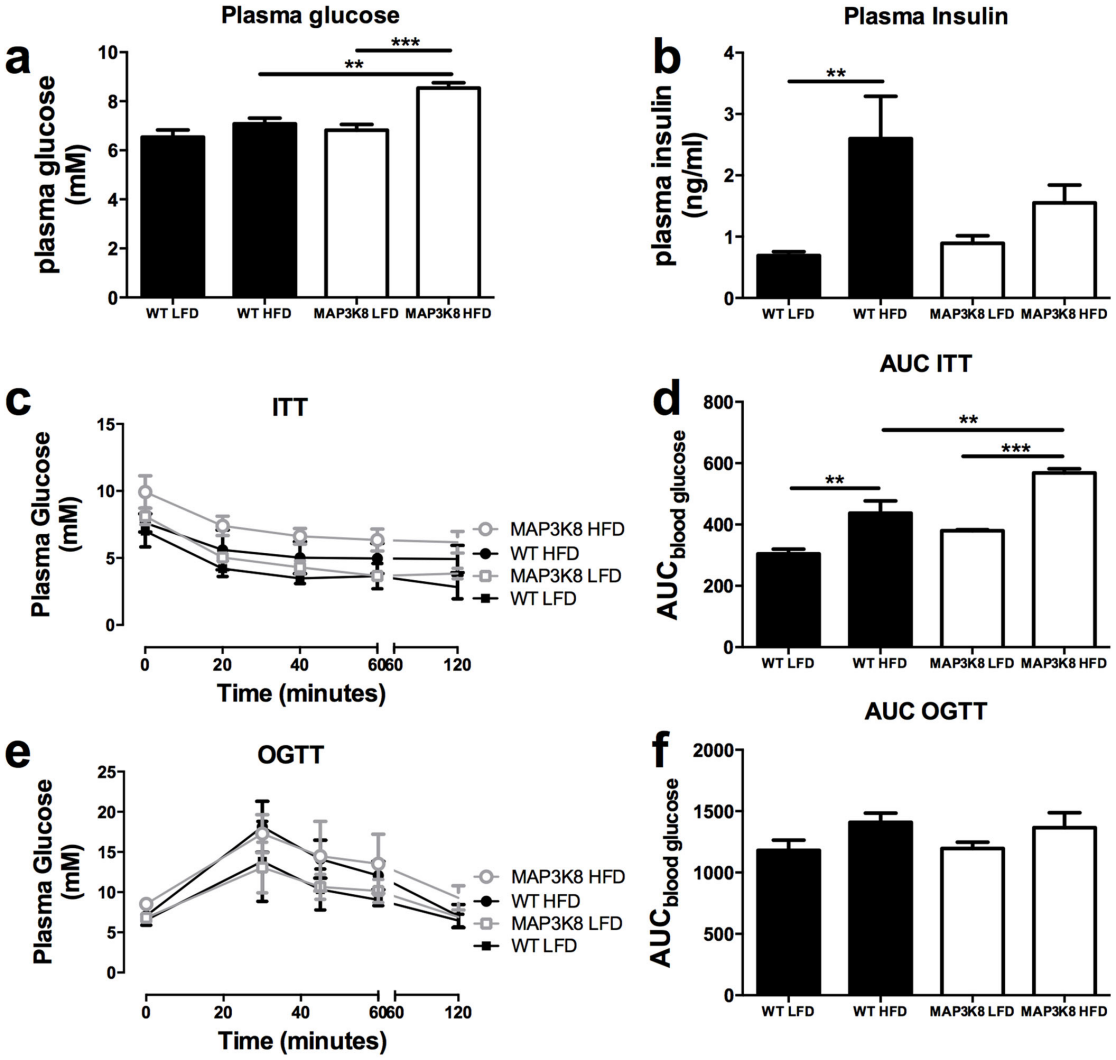
MAP3K8-ko mice display similar bodyweight and insulin sensitivity compared to WT mice. MAP3K8-ko and WT mice were fed a LFD or HFD during 16 weeks. (a) Plasma insulin and (b) plasma glucose levels after diet intervention. Insulin (itt) and oral glucose (ogtt) tolerance tests after 16 weeks of diet intervention. (c) itt after 16 weeks of HFD and (d) area under the curve itt. (e) ogtt after 16 weeks of HFD and (f) area under the curve of ogtt. n = 9 mice per group. * p<0.05, ** p<0.01, *** p<0.001.

Next, we investigated whether the changes in adipose tissue inflammation in MAP3K8-ko and WT mice influence systemic insulin sensitivity. HFD-feeding increased fasting plasma insulin levels in WT, but not MAP3K8-ko mice (**Fig. 5a**). In contrast, basal plasma glucose levels were increased in MAP3K8-ko mice after HFD compared to WT on the same diet (**Fig. 5b**). To investigate whether insulin and glucose homeostasis was different in MAP3K8-ko and WT mice, we subjected the mice to an oral glucose tolerance test (ogtt) and insulin tolerance test (itt). As shown in **Figures 5c–f** MAP3K8 deficiency did not affect glucose or insulin tolerance upon LFD or HFD feeding. Notably, MAP3K8-ko mice fed a HFD even displayed a worsening if insulin tolerance as determined by the area under the curve (AUC), although this finding is partially explained by the elevated basal plasma glucose level.

## Discussion

Inflammation plays a pivotal role in the development of insulin resistance associated with obesity. MAP3K8 (TPL2/COT) has been suggested to be an interesting therapeutic target in order to reduce inflammation, as it regulates activation of NF-κB and JNK transcription factors. Indeed, we observed that the mRNA expression of pro-inflammatory cytokines IL-1ß, IL-6 and IL-8 are associated with expression of MAP3K8 mRNA in human adipose tissue. However, our results do not reveal any association of MAP3K8 expression with markers of insulin sensitivity in human subjects and do not support a crucial role for MAP3K8 as an important regulator in the development of insulin resistance during obesity in mice. The results of this study show that human MAP3K8 adipose tissue expression is positively associated with BMI and expression of several pro-inflammatory cytokines in adipose tissue, but not with systemic inflammatory- or metabolic parameters such as plasma SAA, CRP, insulin or glucose levels. Similarly, although absence of MAP3K8 in mice induced mild changes in inflammation in adipose tissue, there were no differences in systemic insulin resistance even after 16 weeks of a HFD intervention. Therefore, these findings suggest that other inflammatory pathways or kinases may play a more dominant role in the development of obesity induced inflammation and insulin resistance. Hence, MAP3K8 may restrict its role to mediating local cytokine secretion as a downstream kinase of pivotal inflammatory receptors, but without affecting systemic metabolic parameters.

Although MAP3K8 has been reported to be an important regulator of inflammatory pathways in other diseases [14], the effect of MAP3K8 on obesity-induced chronic low-grade inflammation has been a point of debate. Three prior studies have provided contradictory evidence for the role of MAP3K8 in obesity-induced inflammation and metabolic dysfunction [10]–[12]. We found that MAP3K8 is upregulated in human adipose tissue in obese individuals. Moreover, we demonstrate an association between higher MAP3K8 mRNA expression and both IL-1ß and the IL-1ß -responsive cytokines IL-6 and IL-8 in the adipose tissue. MAP3K8 expression levels are not associated with a general enhancement in the inflammatory status of the adipose tissue as mRNA levels of the pro-inflammatory cytokine TNF-α are not changed between the low and high MAP3K8 expressing groups. Although TNF-α is known to be dependent on MAP3K8 in macrophages [9], [15], TNF-α can also be produced independently of MAP3K8 [15], [16]. Moreover, systemic inflammatory markers SAA and CRP were not positively associated with increased MAP3K8 adipose tissue levels, suggestive of a redundant role of MAP3K8 in obesity-induced low-grade systemic inflammation.

Furthermore, we show that MAP3K8-ko and WT mice do not differ in weight after 16 weeks of high fat diet feeding, and that liver weight is similar as well, which is in accordance with earlier studies ([11] [12]). In the present study, the MAP3K8-ko mice did not show ameliorated inflammation in the adipose tissue in response to HFD, which is similar to findings of Lancaster *et al.* but opposed to the study of Perfield *et al*. In fact, expression levels of several macrophage markers (F480, CD68) were higher in adipose tissue of MAP3K8-ko mice compared to WT mice, suggesting increased macrophage infiltration. Interestingly, we observed that expression levels of IL-1ß and IL-1ß effector cytokines (CXCL1 and IL-6) were downregulated, leading to a reduction in circulating plasma CXCL1 levels. Since these cytokines are known to be activated via NFκB and ERK signaling, the reduction in cytokine expression could be secondary to a reduced activation of NFκB or ERK that are downstream molecules of MAP3K8 [14], [16], [17]. However, no difference in activation of NFκB or ERK 1/2 was found, suggesting that these downstream mediators of MAP3K8 were not differently regulated after HFD in both genotypes, hence other molecules downstream of MAP3K8 may be of more importance. The enhanced expression of macrophage markers in absence of MAP3K8 may be a compensatory mechanism for the inhibited cytokine expression by adipose tissue macrophages. Moreover, the increase of TNF-α and IFN-γ expression in adipose tissue in the MAP3K8-ko mice, might explain that no differences are seen on systemic metabolic parameters via compensatory mechanisms, as indicated by unchanged insulin or glucose tolerance in the MAP3K8-ko mice. Together, these results show that absence of MAP3K8 may affect certain inflammatory pathways and macrophage infiltration, but does not affect the presence of crown-like structures or systemic insulin sensitivity. Interestingly, our current results show that insulin levels in the MAP3K8-ko mice are lower, probably explaining a higher fasting glucose level, as was reported before. It may be worthwhile in the future to investigate the effect of MAP3K8 on insulin secretion of the pancreas. Hence, in line with the lower expression levels of IL-1ß that is known to affect beta-cell function [18] the absence of MAP3K8 may affect insulin production.

Parts of our results differ from earlier findings, which may be explained by different experimental or housing conditions. Gut microbiota is suggested to contribute to adipose tissue inflammation and metabolic disease [19]. Therefore, differences in microbiota composition may exist between facilities and could contribute to opposing. In addition, some studies used different types of high fat diets. Another possible explanation is that in the study of Perfield *et al.*, mice were given a high fat diet from 6 weeks of age, while in this study and the study of Lancaster, the mice were several weeks older at the start of the diet intervention. Importantly, in our study, data from mice experiments were in agreement with data derived from human adipose tissue biopsies, confirming a positive association of MAP3K8 expression with local cytokine expression, but not with systemic metabolic parameters.

In summary, these data show that MAP3K8 has a limited role in obesity-induced inflammation and support earlier results by Lancaster *et al.* [12], who did not see protection against obesity-induced metabolic disease in knock-out mice. Altogether, the findings argue against MAP3K8 to be a central kinase in regulating pro-inflammatory signals leading to insulin resistance. For the first time, we show an association between adipose tissue expression of MAP3K8 and IL-1ß, IL-6 and IL-8 in humans. In line with this, our data reveal that MAP3K8 deficiency in HFD-fed mice reduces adipose tissue expression of IL-1ß, IL-6 and IL-8. Therefore, we propose that MAP3K8 may affect production of specific cytokines in adipose tissue inflammation during development of obesity, but that these changes do not translate to profound systemic effects. Although we cannot rule out an effect on insulin sensitivity upon specific inhibition of the MAP3K8 signalling pathway during obesity, future studies should rather illuminate the role of other inflammatory pathways and kinases in adipose tissue affecting systemic metabolic health.

## Supporting Information

Table S1 **List of primers used for RT-PCR.** Primer sequence for all human and mouse genes are listed in this table.
(PDF)
