# Supplementary material for: MAP3K8 (TPL2/COT) Affects Obesity-Induced Adipose Tissue Inflammation without Systemic Effects in Humans and in Mice
Source: PLoS One. 2014 Feb 24;9(2):e89615. doi: 10.1371/journal.pone.0089615 (PMC3933658; doi:10.1371/journal.pone.0089615)
Supplement: Table S1 — List of primers used for RT-PCR. Primer sequence for all human and mouse genes are listed in this table. (PDF) [file pone.0089615.s001.pdf]

Supplemental Table TPL2

Table S1

Gene	*Forward primer*	*Reverse primer*
**m36B4**	AGCGCGTCCTGGCATTGTGTGG	GGGCAGCAGTGGTGGCAGCAGC
**mGAPDH**	AGGTCGGTGTGAACGGATTTG	TGTAGACCATGTAGTTGAGGTCA
**mCD68**	CCAATTCAGGGTGGAAGAAA	CTCGGGCTCTGATGTAGGTC
**mF480**	CTTTGGCTATGGGCTTCCAGTC	GCAAGGAGGACAGAGTTTATCGTG
**mMCP-1**	CCCAATGAGTAGGCTGGAGA	TCTGGACCCATTCCTTCTTG
**mTNF-α**	CAGACCCTCACACTCAGATCATCT	CCTCCACTTGGTGGTTTGCTA
**mIFNγ**	ATGAACGCTACACACTGCATC	CCATCCTTTTGCCAGTTCCTC
**mIL-1β**	GCAACTGTTCCTGAACTCAACT	ATCTTTTGGGGTCCGTCAACT
**mIL-1ra**	AAATCTGCTGGGGACCCTAC	TGAGCTGGTTGTTTCTCAGG
**mIL-6**	CAAGTCGGAGGCTTAATTACACATG	ATTGCCATTGCACAACTCTTTTCT
**mCXCL-1**	TGGCTGGGATTCACCTCAA	GAGTGTGGCTATGACTTCGGTTT
**hB2M**	ATGAGTATGCCTGCCGTGTG	CCAAATGCGGCATCTTCAAAC
**hIL-1ß**	CAGCTACGAATCTCCGACCAC	GGCAGGGAACCAGCATCTTC
**hTNF-α**	TGGCCCAGGCAGTCAGA	GGTTTGCTACAACATGGGCTACA
**hIL-6**	AATTCGGTACATCCTCGACGG	GGTTGTTTTCTGCCAGTGCCT
**hIL-8**	ACTGAGAGTGATTGAGAGTGGAC	AACCCTCTGCACCCAGTTTTC
